# Ultrasonographic evaluation of the common carotid intima-media complex in healthy and overweight/obese children

**DOI:** 10.1590/1677-5449.190003

**Published:** 2019-10-01

**Authors:** Jorge Garcia, Augusto César Garcia Saab Benedeti, Simone Helena Caixe, Francisco Mauad, Carlos Alberto Nogueira-de-Almeida

**Affiliations:** 1 Faculdade de Tecnologia em Saúde – FATESA, Ribeirão Preto, SP, Brasil.; 2 Universidade de São Paulo – USP, Faculdade de Medicina de Ribeirão Preto, Ribeirão Preto, SP, Brasil; 3 Universidade Federal de São Carlos – UFSCAR, Departamento de Medicina, São Carlos, SP, Brasil

**Keywords:** ultrasonography, common carotid, obesity, intima-media complex

## Abstract

**Background:**

Obesity is a global epidemic, including among children. It is therefore necessary to identify cardiovascular changes in overweight/obese children as early as possible. Mode B ultrasonography of the common carotids can be used to precisely evaluate in real time early changes in the thickness of the intima-media complex (IMC), which can detect onset of the atherosclerosis process.

**Objectives:**

This study compared IMC thickness between schoolchildren with and without overweight/obesity.

**Methods:**

A sample of 59 children of both sexes, aged 7 to 10 years, were recruited from health centers in São Paulo, Brazil. Children were classified by z scores for body mass index (BMI) into two groups, with or without overweight/obesity. These groups were then compared in terms of IMC thickness.

**Results:**

The groups were homogenous for age and sex. The mean IMC measurement in the group with overweight/obesity was 0.49 (± 0.07) mm, whereas in the group free from overweight/obesity it was 0.41 (± 0.05) mm (p < 0.01). These differences were maintained when groups with and without overweight/obesity were compared separately by sex and for right and left sides. The coefficient for the correlation between IMC measurement and BMI z score was 0.61 (95% confidence interval = 0.42-0.75). Within the same nutritional status group, there were no differences between sexes or between right and left sides.

**Conclusions:**

Intima-media thickness was greater among children with overweight/obesity and was directly proportional to BMI z score, denoting increased cardiovascular risk in this group.

## INTRODUCTION

Over recent decades, childhood obesity has increased to the point that it is considered a global epidemic and one of the major public health challenges in both first world and developing countries.^1^^-^ 3 Studies have demonstrated striking increases in the rates of obesity, the prevalence of which tripled in countries such as Australia, Brazil, Canada, Chile, Finland, France, Germany, Greece, Japan, the United Kingdom, and the United States over the period between the start of the 1970s and end of the 1990s.^1^^,^ 3

Developing countries (and Brazil) are going through an epidemiological transition, with non-transmissible chronic diseases predominating over transmissible diseases, and a nutritional transition, with progressively increasing obesity taking the place of malnutrition.^4^ Additionally, studies suggest that maintenance of obesity is directly associated with morbidity and mortality from cardiovascular diseases.^5^^-^ 9

In Brazil, the Ministry of Health’s strategic action plan for non-transmissible chronic diseases (2011-2022) states that the prevalence of overweight among children aged 5 to 9 years has reached 33.5%, while obesity in the same age group has reached 14.3%, according to the Brazilian Institute of Geography and Statistics (IBGE - Instituto Brasileiro de Geografia e Estatística).^10^^,^ 11 In the 10 to 19 years age group, the prevalence of overweight among adolescents was 20%, while the prevalence of obesity was 4% among girls and 5.9% among boys.^10^ Furthermore, according to the 2008-2009 Family Budgets Survey (POF - Pesquisa de Orçamentos Familiares), the highest rates of overweight and obesity were observed in the South and Southeast administrative regions in all age groups studied and in both sexes.^10^ The increase in overweight and obesity was even more significant in the 5 to 9 years age group.^10^

There is evidence to show that atherosclerosis starts in childhood with build up of lipids in the arterial intima,^7^ which establishes a clear relationship between obesity and cardiovascular disease.^12^ Longitudinal studies have shown associations between excess weight during the first 10 years of life and high rates of morbidity and mortality from cardiovascular diseases in adulthood.^13^^,^ 14 Histologically, increased thickness of the intima-media complex (IMC) comprises hypertrophy of the medial (muscular) and intimal (endothelium) layers of the artery wall. Intima-media thickening is considered a noninvasive and early marker of atherosclerosis. It can reflect increased cardiovascular risk and is associated with higher risk of acute myocardial infarction and/or stroke.^15^

Structural evidence of early atherosclerosis is often found in adolescents and young adults when their arteries are examined in autopsies. In these cases, the extent of lesions increases with age and with the number and severity of traditional cardiovascular risk factors.^16^^,^ 17

Obesity is associated with atherosclerosis and leads to development of cardiovascular changes^18^ that should be assessed as early as possible. Ultrasonography of the common carotids can be used to conduct this assessment adequately and safely, by measuring the IMC thickness, which is an early subclinical marker of the atherogenesis that can be used to compare healthy children and adolescents who are or are not obese. This study evaluated common carotid IMC thickness using the ultrasonographic method, in children with overweight/obesity and compared them with an equivalent group of children with healthy weights.

## MATERIALS AND METHODS

This observational, cross-sectional, comparative study was conducted with 59 children of both sexes, stratified according to nutritional status into two groups: healthy weight and overweight/obese with no associated comorbidities. The sample was recruited at primary care health centers from the population of São Paulo and other towns in the region. Data were collected from May 2013 to October 2014.

The sample comprised 59 children of both sexes aged from 7 to 10 years (mean of 8.8 years), distributed into two groups depending on presence or absence of overweight/obesity. In order to classify overweight/obesity, weight and height were measured and used to calculated body mass index (BMI) according to the international recommendations.^19^^,^ 20 Nutritional status was classified by BMI z scores. Values from -2 to +1 were classed as healthy weight; from +1 to +2 as overweight; and greater than +2, as obesity.^4^^,^ 19 Participants with BMI z scores greater than +1 took part in the study, taking together overweight and obesity, as assessed with the World Health Organization (WHO) AnthroPlus software program, using the WHO reference curves.^19^^,^ 21

The data collection protocol covered weight and height measurements. A Kratos-Cas electronic balance was used to measure weight and a Kratos-Cas portable anthropometer was used to measure height.

Mode B ultrasound scans of the common carotid IMC were performed using an ESAOTE Healthcare My Lab 70 XVG, with a flat (linear) transducer with frequency variable from 7.5 to 12 Mhz and non-ionic aqueous gel. The scanner is equipped with a high-resolution analytical system (grayscale).

Mode B ultrasonography was conducted without prior knowledge of the children’s nutritional status. They were only stratified by nutritional status after data had been collected and tabulated, into two groups: healthy weight (BMI z score from -2 to +1) and overweight/obese (BMI z score exceeding +1). A statistical analysis was conducted to verify whether the groups were homogenous or heterogeneous in terms of distribution by age, sex, and presence or absence of overweight/obesity. For acquisition of ultrasound images, children were examined in the supine position, with neck extended and the head rotated laterally through 45 degrees in the direction contralateral to the artery being examined (with no pillow), by an examiner positioned behind the patient’s head.^15^

**Figure 1:**
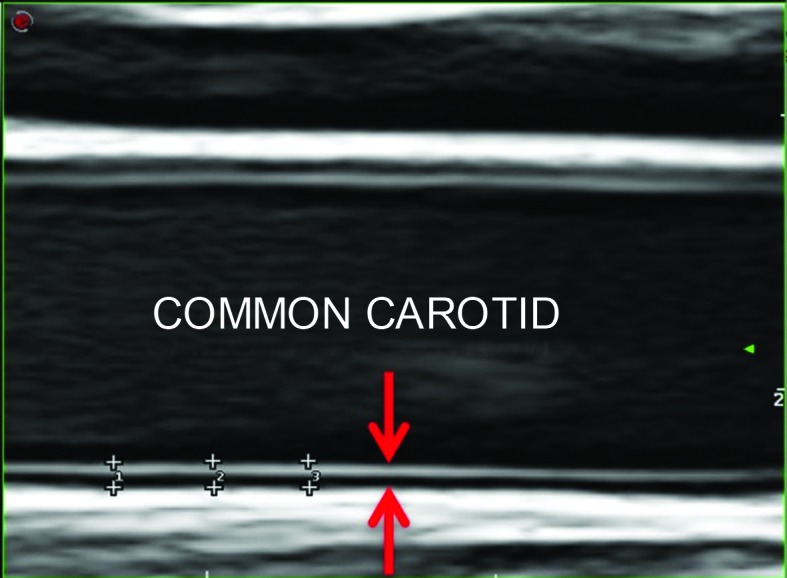
Quantification of the intima-media complex of the common carotid artery (electronic markers: +). Intima-media complex of the common carotid (arrows).

The IMC of the common carotid arteries was measured in mode B by a single observer, at the distal third up to a point 2 cm from the bifurcation, with an angle of incidence of 90º, at the posterior wall of the carotid image.^15^ The IMC measurement was defined as the distance between two echogenic lines; the lumen-intima interface and the media-adventitial interface. Three manual measurements were taken of each common carotid (as shown in Figure 1) and the mean calculated.^15^^,^ 22

Data are expressed as mean ± standard deviation. The mixed-effects linear regression model was employed.^23^ The research project was duly approved by the Human Research Ethics Committee, under decision number 215.788.

## RESULTS

The overweight/obesity group comprised 29 children (49.2%), 16 of whom were male (55.2%) and 13 of whom were female (44.8%). The healthy weight group was comprised of 30 children (50.8%), 12 of whom were male (40.0%) and 18 of whom were female (60.0%).

Table 1 lists general characteristics of the groups: sex, age, and BMI z score, by nutritional status. Mean age was similar in both groups (children with overweight/obesity: 8.9 ± 1.7 years; children with healthy weight: 8.7 ± 1.1 years), as was sex (males: 28 and Females: 31).

**Table 1:** General characteristics of the groups: sex, age, and z score for body mass index, by nutritional status.

	**Overweight/obesity** **(n = 29)**	**Healthy weight** **(n = 30)**	**p**
Boys	16 (55.2%)	12 (40%)	0.24
Girls	13 (44.8%)	18 (60%)	
Age (months)	107 ± 12 (Mean ± SD)	105 ± 13 (Mean ± SD)	0.52
BMI z score	3.2 ± 1.1 (Mean ± SD)	0.16 ± 0.9 (Mean ± SD)	< 0.01

BMI = body mass index; SD = standard deviation.

Mean IMC thickness for right and left side common carotids was 0.5 mm and 0.49 mm, respectively (mean: 0.49 mm) in the group with overweight/obesity and 0.41 mm on both sides (mean: 0.41 mm) in the healthy weight group (Tables 2 and 3). These data confirm that the groups were, initially, only different in terms of what was being tested, i.e. overweight/obesity and IMC, being similar in respect of the other personal variables studied (age, sex, common carotid on the right and left sides).

**Table 2:** Distribution of the variables listed by nutritional status, sex, and right and left side common carotid correlated with intima-media complex thickness.

**Group**	**Side/Sex**	**Total**	**mean IMC (mm)**	**SD**	**Minimum**	**1^st^ quartile**	**Median**	**3^rd^ quartile**	**Maximum**
Healthy weight	R + L	60	0.41	0.05	0.40	0.40	0.44	0.40	0.53
Overweight/obesity	R + L	58	0.49	0.07	0.03	0.48	0.50	0.53	0.60
Healthy weight	R	30	0.41	0.06	0.30	0.40	0.40	0.47	0.50
Healthy weight	L	30	0.41	0.04	0.36	0.40	0.40	0.40	0.53
Overweight/obesity	R	29	0.5	0.07	0.30	0.50	0.50	0.53	0.60
Overweight/obesity	L	29	0.49	0.07	0.30	0.48	0.50	0.50	0.60
Healthy weight	M	24	0.42	0.06	0.30	0.40	0.40	0.40	0.50
Healthy weight	F	36	0.41	0.04	0.30	0.40	0.40	0.41	0.53
Overweight/obesity	M	32	0.50	0.07	0.40	0.48	0.50	0.54	0.60
Overweight/obesity	F	26	0.48	0.07	0.30	0.48	0.50	0.50	0.57

IMC = intima-media complex; R = right; L = left; M = male; F = female; SD = standard deviation.

**Table 3:** Comparison of intima-media complex thickness, by nutritional status, sex, and right and left side common carotids.

**Comparison**	**Estimated difference (mm)**	**95% confidence interval**	**p**
Overweight/obesity – Healthy weight	0.80	0.05-0.11	< 0.01
R side – L side	0.00	-0.01-0.01	0.94
Female – Male	-0.01	-0.04-0.02	0.47
Overweight/obesity – Healthy weight (R side)	0.08	0.05-0.11	< 0.01
Overweight/obesity – Healthy weight (L side)	0.07	0.04-0.10	< 0.01
Overweight/obesity – Healthy weight (R side – L side)	0.00	-0.01-0.02	0.60
Healthy weight – (R side – L side)	0.00	-0.02-0.01	0.67
Overweight/obesity – Healthy weight (Female)	0.06	0.02-0.10	< 0.01
Overweight/obesity – Healthy weight (Male)	0.09	0.05-0.14	< 0.01
Overweight/obesity (Female – Male)	-0.03	-0.07-0.02	0.21
Healthy weight (Female – Male)	0.00	-0.04-0.05	0.83

R = right; L = left.

Table 2 lists descriptive statistics for IMC thickness of the common carotids on both sides, measured in the groups with presence or absence of overweight/obesity. Children classified with overweight/obesity had thicker common carotid IMC than those in the healthy weight group, with a mean of 0.49 mm in the group overweight/obesity and 0.41 mm in the healthy weight group (Table 2), with p < 0.01. There was no difference between the IMC measured in the right and left common carotids (p = 0.94). Mean IMC thickness in the common carotids of the group with overweight/obesity were 0.49 mm on the left side and 0.5 mm on the right side (p = 0.60), while the value for IMC thickness in the healthy weight group was 0.41 mm on both sides (p = 0.67). Mean IMC thickness was similar in both sexes (p = 0.47).

**Figure 2:**
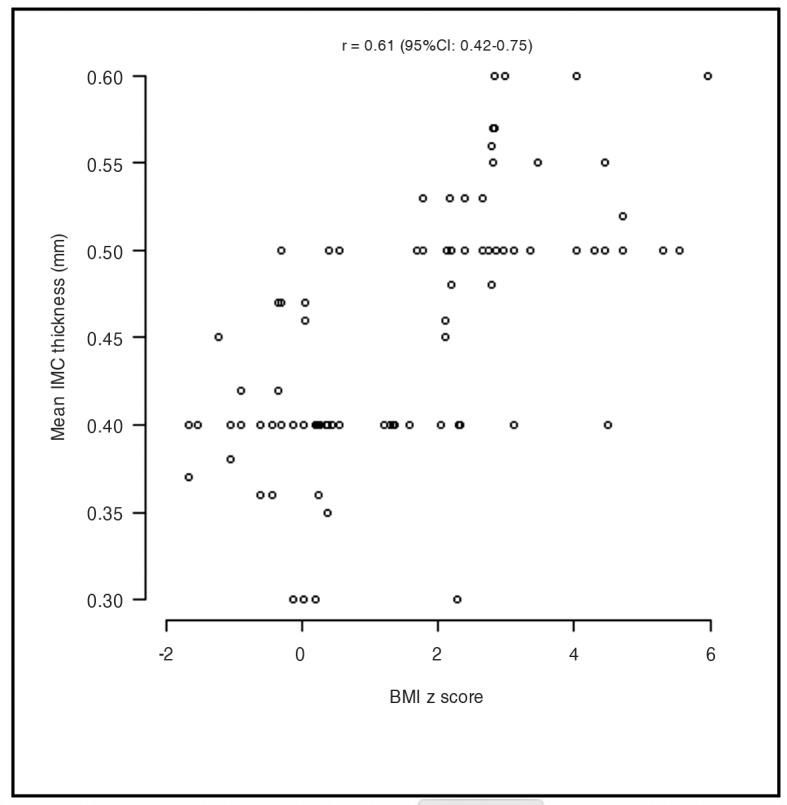
Correlation between z score for body mass index (BMI) and intima-media complex (IMC) thickness. Pearson’s correlation coefficient: r = 0.61, 95% confidence interval = 0.42-0.75.

The correlation between BMI z score and IMC was investigated using Pearson’s correlation coefficient (r), which quantifies associations between quantitative variables. The correlation coefficient was 0.61, with statistical significance (p < 0.01) and a 95% confidence interval of 0.42-0.75. Figure 2 illustrates a strong positive correlation in both groups of study subjects, by degree of common carotid IMC thickness, according to the mixed effects linear regression model.^23^

## DISCUSSION

Children with excess weight are exposed to cardiovascular risk factors as early as the prepubescent phase, predisposing to endothelial dysfunction and contributing to increased IMC thickness in the common carotids.^24^ In 2010, Juonala et al.^25^ analyzed data from four large population and cohort studies (the Muscatine Study, the Cardiovascular Risk in Young Finns Study, the Bogalusa Heart Study, and the Childhood Determinants of Adult Health Study), showing that exposure to multiple risk factors from 9 years of age onward was predictive of subclinical atherosclerosis in adulthood. They therefore suggested that these factors, which are directly associated with morbidity and mortality due to cardiovascular disease, should be measured from 9 years of age onwards.

Intima-media thickness is a well-established subclinical marker of atherosclerosis and can also be indicative of future cardiovascular disease.^26^ The present study confirms the ultrasonographic findings of IMC changes in children with overweight/obesity (Tables 2 and 3), when compared to a control group, irrespective of sex or of whether the right or left side common carotid is measured. A case-control study conducted in Belgium by Beauloye et al.^27^ utilized ultrasonography to evaluate the IMC thickness of healthy obese and not obese individuals aged from 8 to 18 years (mean of 12.7 and 13 years, respectively). The mean values for IMC thickness were 0.438 mm in controls and 0.470 mm in obese subjects, which is a statistically significant difference (p = 0.0031). Although mean age was older than in the present study, the results were similar in terms of the significant difference in IMC thickness between the two groups. It should be noted that, considering the two studies as a sequence, these changes in the carotid have early onset and persist throughout childhood and adolescence. These results are similar to those of other studies that report greater IMC thickness in obese children and adolescents when compared to control groups.^28^^,^ 29 Fang et al.^30^ evaluated 86 obese children and adolescents of both sexes distributed into two groups: 23 obese children with metabolic syndrome (mean age: 10.9 ± 1.6 years) and 63 obese children without metabolic syndrome (mean age: 10.5 ± 1.6 years). A control group comprised 22 non-obese healthy children and adolescents (mean age: 11.1 ± 2.1 years). The obese groups, with and without metabolic syndrome, both exhibited increased IMC thickness when compared to the control group.

The present study is subject to certain limitations. The most important is the cross-sectional study design, which precludes use of the results for cause and effect analyses. The total number of children assessed was also small, although adequate for the statistical analyses. Finally, a convenience sample was used, preventing extrapolation of the results to the population of children in this age group.

The results observed allow for the conclusion that children with overweight/obesity exhibited thicker IMC in the common carotids (0.49 ± 0.07 mm) when compared to healthy weight children (0.41 ± 0.05 mm) of the same age group and of both sexes (p < 0.01), in line with results observed by other authors.^31^^-^ 33 The most important contribution of this study is related to the age group studied, since ultrasonographic studies of the IMC in subjects of this age are very rare. The results shown will undoubtedly contribute to better understanding of the atherosclerotic process in children with excess weight.

## References

[1] Han JC, Lawlor DA, Kimm SYS (2010). Childhood obesity. Lancet.

[2] Mutangadura G (2004). World Health Report 2002: Reducing Risks, Promoting Healthy Life World Health Organization, Geneva, 2002, 250 pages, US$ 13.50, ISBN 9-2415-6207-2. Agric Econ.

[3] Wang Y, Lobstein T (2006). Worldwide trends in childhood overweight and obesity. Int J Pediatr Obes.

[4] Sociedade Brasileira de Pediatria (2008). Obesidade na infância e adolescência: manual de orientação..

[5] Caixe SH, Benedeti ACGS, Garcia J (2014). Evaluation of echocardiography as a marker of cardiovascular risk in obese children and adolescents. International Journal of Clinical Pediatrics..

[6] Cercato C, Silva S, Sato A, Mancini M, Halpern A (2000). Risco cardiovascular em uma população de obesos. Arq Bras Endocrinol Metabol.

[7] Costa KCM, Ciampo LAD, Silva PS, Lima JC, Martins WP, Nogueira-de-Almeida CA (2018). Ultrasonographic markers of cardiovascular disease risk in obese children. Rev Paul Pediatr.

[8] Marchi-Alves LM, Yagui CM, Rodrigues CS, Mazzo A, Rangel EML, Girão FB (2011). Obesidade infantil ontem e hoje: importância da avaliação antropométrica pelo enfermeiro. Esc Anna Nery.

[9] Nogueira-de-Almeida CA, Mello ED (2018). Correlation of body mass index Z-scores with glucose and lipid profiles among overweight and obese children and adolescents. J Pediatr (Rio J).

[10] IBGE (2010). Pesquisa de Orçamentos Familiares 2008-2009: Antropometria e estado nutricional de crianças, adolescentes e adultos no Brasil.

[11] Malta DC, Morais OL, Silva JB (2011). Apresentação do plano de ações estratégicas para o enfrentamento das doenças crônicas não transmissíveis no Brasil, 2011 a 2022. Epidemiol Serv Saude.

[12] McGill HC (1984). George Lyman Duff memorial lecture. Persistent problems in the pathogenesis of atherosclerosis. Arterioscler Thromb Vasc Biol.

[13] Must A, Jacques PF, Dallal GE, Bajema CJ, Dietz WH (1992). Long-Term morbidity and mortality of overweight adolescents. N Engl J Med.

[14] Oliveira CL, Mello MT, Cintra IP, Fisberg M (2004). Obesidade e síndrome metabólica na infância e adolescência. Rev Nutr.

[15] Touboul PJ, Hennerici MG, Meairs S (2006). Mannheim carotid intima-media thickness consensus (2004-2006). Cerebrovasc Dis.

[16] AMA (1990). Relationship of atherosclerosis in young men to serum lipoprotein cholesterol concentrations and smoking. JAMA.

[17] McGill HC, McMahan CA, Herderick EE (2000). Effects of coronary heart disease risk factors on atherosclerosis of selected regions of the aorta and right coronary artery. Arterioscler Thromb Vasc Biol.

[18] Nogueira-de-Almeida CA, Caixe SH, Benedeti ACGS, Garcia J (2016). Echocardiography evaluation as a marker of cardiovascular risk on obese children and adolescents. The FASEB Journal.

[19] Sellen D (1998). Physical Status: The use and interpretation of Anthropometry. Report of a WHO Expert Committee. WHO Technical Report Series No. 854. Pp. 452. (WHO, Geneva, 1995.) Swiss Fr 71.00. J Biosoc Sci.

[20] de Almeida CAN, Ricco RG (1998). Avaliação do estado nutricional com ênfase à antropometria. Pediatria.

[21] WHO (2010). WHO Anthro for personal computers, version 3.2. 2, 2011: software for assessing growth and development of the world’s children..

[22] Pignoli P, Tremoli E, Poli A, Oreste P, Paoletti R (1986). Intimal plus medial thickness of the arterial wall: a direct measurement with ultrasound imaging. Circulation.

[23] Schall R (1991). Estimation in generalized linear models with random effects. Biometrika.

[24] Raitakari OT, Juonala M, Kähönen M (2003). Cardiovascular risk factors in childhood and carotid artery intima-media thickness in adulthood. JAMA.

[25] Juonala M, Magnussen CG, Venn A (2010). Influence of age on associations between childhood risk factors and carotid intima-media thickness in adulthood clinical perspective: the Cardiovascular Risk in Young Finns Study, the Childhood Determinants of Adult Health Study, the Bogalusa Heart Study, and the Muscatine Study for the International Childhood Cardiovascular Cohort (i3C) Consortium. Circulation.

[26] Lorenz MW, Markus HS, Bots ML, Rosvall M, Sitzer M (2007). Prediction of clinical cardiovascular events with carotid intima-media thickness: A systematic review and meta-analysis. Circulation.

[27] Beauloye V, Zech F, Tran HT, Clapuyt P, Maes M, Brichard SM (2007). Determinants of early atherosclerosis in obese children and adolescents. J Clin Endocrinol Metab.

[28] Atabek ME, Pirgon O, Kivrak AS (2007). Evidence for association between insulin resistance and premature carotid atherosclerosis in childhood obesity. Pediatr Res.

[29] Giannini C, de Giorgis T, Scarinci A (2008). Obese related effects of inflammatory markers and insulin resistance on increased carotid intima media thickness in pre-pubertal children. Atherosclerosis.

[30] Fang J, Zhang JP, Luo CX, Yu XM, Lv LQ Carotid Intima-media thickness in childhood and adolescent obesity relations to abdominal obesity, high triglyceride level and insulin resistance. Int J Med Sci..

[31] Gustiene O, Slapikas R, Marcinkeviciene J (2005). Relationship between the metabolic syndrome, endothelial function and intima-media thickness in asymptomatic middle-aged individuals. Medicina.

[32] Iannuzzi A, Licenziati MR, Acampora C (2004). Increased carotid intima-media thickness and stiffness in obese children. Diabetes Care.

[33] Zhu W, Huang X, He J, Li M, Neubauer H (2005). Arterial intima-media thickening and endothelial dysfunction in obese Chinese children. Eur J Pediatr.

