# Supplementary material for: Ultrasonographic evaluation of the common carotid intima-media complex in healthy and overweight/obese children
Source: J Vasc Bras. 2019 Oct 1;18:e20190003. doi: 10.1590/1677-5449.190003 (PMC6788858; doi:10.1590/1677-5449.190003)
Supplement: Translation

# Translation: Avaliação ultrassonográfica do complexo médio-intimal das carótidas comuns em crianças eutróficas e portadoras de sobrepeso/obesidade

**Authors:** Jorge Garcia, Augusto César Garcia Saab Benedeti, Simone Helena Caixe, Francisco Mauad, Carlos Alberto Nogueira-de-Almeida

**Affiliations:** 1 Faculdade de Tecnologia em Saúde – FATESA, Ribeirão Preto, SP, Brasil.; 2 Universidade de São Paulo – USP, Faculdade de Medicina de Ribeirão Preto, Ribeirão Preto, SP, Brasil; 3 Universidade Federal de São Carlos – UFSCAR, Departamento de Medicina, São Carlos, SP, Brasil

**Keywords:** ultrassonografia, carótida comum, obesidade, complexo médio-intimal

## Abstract

**Contexto:**

A obesidade é uma epidemia global, inclusive entre as crianças. Diante desse perfil, torna-se necessário identificar precocemente alterações cardiovasculares presentes em crianças com sobrepeso/obesidade. A ultrassonografia no modo B das carótidas comuns avalia, com precisão e em tempo real, as alterações precoces na medição da espessura do complexo médio-intimal (CMI), podendo detectar o início do processo de aterosclerose.

**Objetivos:**

O presente estudo comparou a espessura do CMI entre crianças escolares com e sem sobrepeso/obesidade.

**Métodos:**

Foram incluídas 59 crianças de ambos os sexos, entre 7 e 10 anos de idade, oriundas de centros de saúde de São Paulo. As crianças foram caracterizadas de acordo com o escore z do índice de massa corporal (IMC) em dois grupos, com e sem sobrepeso/obesidade. Os grupos foram comparados em relação à espessura do CMI.

**Resultados:**

Os grupos foram homogêneos em idade e sexo. A medida média do CMI no grupo com sobrepeso/obesidade foi de 0,49 (± 0,07) mm; no grupo não sobrepeso/obeso, foi de 0,41 (± 0,05) mm (p < 0,01). Essas diferenças se mantiveram quando os grupos com e sem sobrepeso/obesidade foram comparados separadamente por sexo e pelos lados direito e esquerdo. O coeficiente de correlação entre a medida do CMI e o escore z do IMC foi de 0,61 (intervalo de confiança de 95% = 0,42-0,75). Dentro do mesmo estado nutricional, não houve diferença entre os gêneros, nem entre os lados direito e esquerdo.

**Conclusões:**

A espessura do CMI de crianças com sobrepeso/obesidade foi maior e diretamente proporcional ao escore z do IMC, denotando maior risco cardiovascular nesse grupo.

## INTRODUÇÃO

Nas últimas décadas, a obesidade infantil vem sendo considerada uma epidemia global e um dos principais desafios de saúde pública tanto nos países de primeiro mundo como nos países em desenvolvimento^1^^-^ 3. Estudos têm evidenciado um aumento expressivo da obesidade, cuja prevalência triplicou em países como Austrália, Brasil, Canadá, Chile, Finlândia, França, Alemanha, Grécia, Japão e Reino Unido e Estados Unidos, no período compreendido entre os primeiros anos da década de 1970 e os últimos anos da década de 1990^1^^,^ 3.

Vive-se hoje, nos países em desenvolvimento e no Brasil, uma transição epidemiológica, com predominância de doenças crônicas não transmissíveis em relação às transmissíveis, associada à transição nutricional, com aumento progressivo da obesidade no lugar da desnutrição^4^. Além disso, alguns estudos sugerem que a manutenção da obesidade está diretamente associada à morbimortalidade por doenças cardiovasculares^5^^-^ 9.

No Brasil, segundo os planos de ações estratégicas para o enfrentamento das doenças crônicas não transmissíveis do Ministério de Saúde (2011-2022), a prevalência de sobrepeso em crianças na faixa etária de cinco a nove anos de idade atingiu 33,5%, enquanto a obesidade, nessa mesma faixa etária, chegou a 14,3% (IBGE)^10^^,^ 11. Nos indivíduos da faixa etária de 10 a 19 anos, a prevalência de sobrepeso foi de 20% nos adolescentes, enquanto a prevalência de obesidade foi de 4% nas meninas e de 5,9% nos meninos, segundo o Instituto Brasileiro de Geografia e Estatística (IBGE)^10^. Além disso, segundo a Pesquisa de Orçamentos Familiares (POF 2008-2009), nas regiões Sul e Sudeste ocorreram as maiores taxas de sobrepeso e obesidade em todas as faixas etárias estudadas para ambos os sexos (IBGE)^10^. Na faixa etária entre 5 e 9 anos, houve incremento ainda mais significativo de sobrepeso e obesidade (IBGE)^10^.

Evidências indicam que a aterosclerose começa na infância com acúmulo de lipídios na íntima das artérias^7^, o que estabelece uma relação estreita entre obesidade e doença cardiovascular^12^. Em estudos longitudinais, têm-se mostrado associação entre o excesso de peso nas primeiras décadas de vida e a alta taxa de morbimortalidade na vida adulta por doenças cardiovasculares^13^^,^ 14. O aumento da espessura do complexo médio-intimal (CMI) é constituído histologicamente pela hipertrofia das camadas média (muscular) e íntima (endotélio) da parede arterial e é considerado marcador não invasivo e precoce da aterosclerose. Pode refletir o aumento do risco cardiovascular, sendo associado a maior risco de infarto agudo do miocárdio e/ou de acidente vascular cerebral^15^.

Evidência estrutural de aterosclerose precoce é comumente encontrada em adolescentes e adultos jovens quando suas artérias são examinadas em autópsias. Nesses casos, observa-se um aumento da extensão das lesões conforme o aumento da idade e o número e agravamento dos tradicionais fatores de risco cardiovascular^16^^,^ 17.

A obesidade está associada à aterosclerose e pode cursar com alterações cardiovasculares^18^ que precisam ser precocemente avaliadas. A ultrassonografia das carótidas comuns realiza essa avaliação de forma adequada e segura, através da medida da espessura do CMI, marcador subclínico precoce da aterogênese que pode ser comparado entre crianças e adolescentes saudáveis, obesos e não obesos. O presente estudo buscou avaliar a espessura do CMI de carótidas comuns, através do método ultrassonográfico, em crianças com sobrepeso/obesidade em comparação com um grupo eutrófico equivalente.

## MATERIAIS E MÉTODOS

Este estudo observacional, transversal, comparativo de dois grupos, foi realizado com 59 crianças de ambos os sexos, estratificadas de acordo com o estado nutricional em dois grupos: eutróficos e portadores de sobrepeso/obesidade, sem comorbidades associadas. A amostra foi composta por indivíduos originários das unidades básicas de saúde da população de São Paulo e de diferentes cidades da região. A coleta foi realizada no período de maio de 2013 a outubro de 2014.

A casuística consistiu em 59 crianças, de ambos os sexos, com idades entre 7 e 10 anos (média de 8,8 anos), distribuídas em dois grupos conforme a presença ou não de sobrepeso/obesidade. Para avaliação de sobrepeso/obesidade, foram realizadas medidas de peso e estatura e foi calculado o índice de massa corporal (IMC) de acordo com as recomendações internacionais^19^^,^ 20. A classificação da condição nutricional foi efetuada pela determinação da posição em relação ao escore z do IMC. Valores entre -2 e +1 significam eutrofia; entre +1 e +2, sobrepeso; e acima de +2, obesidade^4^^,^ 19. Os grupos sobrepeso e obesidade foram considerados em conjunto. Participaram do estudo apenas aqueles com escore z do IMC acima de -2, avaliados através do programa de computador WHO AnthroPlus, usando curvas de referência da Organização Mundial da Saúde (WHO)^19^^,^ 21.

O protocolo de coleta de dados compreendeu medida de peso e altura. Uma balança eletrônica da marca Kratos-Cas foi utilizada para aferição do peso e um antropômetro portátil Kratos-Cas para aferição da altura.

Para a realização das avaliações ultrassonográficas no modo B do CMI das carótidas comuns, utilizou-se um equipamento da marca ESAOTE Healthcare, modelo My Lab 70 XVG, com transdutor plano (linear) de frequência variável de 7,5 e 12 Mhz e uso de gel aquoso não iônico. O equipamento é provido de sistema analítico de alta resolução de imagem (escala de cinza).

A ultrassonografia no modo B foi realizada sem conhecimento prévio do estado nutricional das crianças. Somente após terem sido coletados e tabulados os dados, as crianças foram estratificadas de acordo com o estado nutricional em dois grupos: eutróficas (escore z do IMC entre -2 e +1) e sobrepeso/obesas (escore z do IMC superior a +1). A avaliação estatística foi conduzida a fim de verificar se os grupos eram homogêneos ou heterogêneos em relação à distribuição etária, ao sexo e à presença ou ausência de sobrepeso/obesidade. Para aquisição das imagens, as crianças foram examinadas na posição supina, com extensão do pescoço e rotação lateral da cabeça de 45 graus para o lado contrário à artéria examinada (sem travesseiro) e o examinador posicionado atrás da cabeça do paciente^15^.

A mensuração do CMI das artérias carótidas comuns foi realizada no modo B por um único observador, no seu 1/3 distal até chegar a 2 cm da bifurcação, com ângulo de insonação de 90º, na parede posterior da imagem da carótida^15^. A medição do CMI foi definida como a distância entre duas linhas ecogênicas, a interface lúmen-íntima e média-adventícia. Foram realizadas três medições manuais das carótidas comuns, conforme pode ser visto na Figura 1, permitindo o cálculo da média^15^^,^ 22.

**Figura 1:** Quantificação do complexo médio-intimal de artéria carótida comum (marcador eletrônico +). Complexo médio-intimal da carótida comum (setas).

Os dados estão apresentados como média ± desvio padrão. Foi utilizado o modelo de regressão linear com efeitos mistos^23^. O projeto de pesquisa foi devidamente aprovado pelo Comitê de Ética em Pesquisa envolvendo seres humanos, com parecer número 215.788.

## RESULTADOS

O grupo de sobrepeso/obesidade foi composto por 29 crianças (49,2%), sendo 16 do sexo masculino (55,2%) e 13 do sexo feminino (44,8%). O grupo eutrófico foi composto por 30 crianças (50,8%), sendo 12 do sexo masculino (40,0%) e 18 do sexo feminino (60,0%).

A Tabela 1 apresenta as características gerais dos grupos por sexo, idade e escore z do IMC, de acordo com o estado nutricional. A média de idade foi semelhante nos dois grupos (crianças com sobrepeso/obesidade: 8,9 ± 1,7 anos; crianças eutróficas: 8,7 ± 1,1 anos), assim como o sexo (masculino: 28 e feminino: 31).

**Tabela 1:** Características gerais dos grupos listadas por sexo, idade e escore z do índice de massa corporal de acordo com o estado nutricional.

	**Sobrepeso/obesidade** **(n = 29)**	**Eutrofia** **(n = 30)**	**p**
Meninos	16 (55,2%)	12 (40%)	0,24
Meninas	13 (44,8%)	18 (60%)	
Idade (meses)	107 ± 12 (Média ± DP)	105 ± 13 (Média ± DP)	0,52
Escore z do IMC	3,2 ± 1,1 (Média ± DP)	0,16 ± 0,9 (Média ± DP)	< 0,01

IMC = índice de massa corporal; DP = desvio padrão.

A medida da espessura do CMI no lado direito e esquerdo das carótidas comuns foi de 0,5 mm e 0,49 mm respectivamente (média: 0,49 mm) no grupo com sobrepeso/obesidade e de 0,41 mm em ambos os lados (média: 0,41 mm) no grupo eutrófico (Tabelas 2 e 3). Esses dados confirmam que os grupos eram, inicialmente, diferentes apenas em relação ao que se estava testando, ou seja, a sobrepeso/obesidade e o CMI, sendo semelhantes nas demais variáveis pessoais estudadas (idade, sexo, carótida comum nos lados direito e esquerdo).

**Tabela 2:** Distribuição das variáveis listadas por estado nutricional, sexo e lados direito e esquerdo da carótida comum correlacionados com o complexo médio-intimal.

**Grupo**	**Lado/sexo**	**Total**	**CMI média (mm)**	**DP**	**Mínimo**	**1º quartil**	**Mediana**	**3º quartil**	**Máximo**
Eutrofia	D + E	60	0,41	0,05	0,40	0,40	0,44	0,40	0,53
Sobrepeso/obesidade	D + E	58	0,49	0,07	0,03	0,48	0,50	0,53	0,60
Eutrofia	D	30	0,41	0,06	0,30	0,40	0,40	0,47	0,50
Eutrofia	E	30	0,41	0,04	0,36	0,40	0,40	0,40	0,53
Sobrepeso/obesidade	D	29	0,5	0,07	0,30	0,50	0,50	0,53	0,60
Sobrepeso/obesidade	E	29	0,49	0,07	0,30	0,48	0,50	0,50	0,60
Eutrofia	M	24	0,42	0,06	0,30	0,40	0,40	0,40	0,50
Eutrofia	F	36	0,41	0,04	0,30	0,40	0,40	0,41	0,53
Sobrepeso/obesidade	M	32	0,50	0,07	0,40	0,48	0,50	0,54	0,60
Sobrepeso/obesidade	F	26	0,48	0,07	0,30	0,48	0,50	0,50	0,57

CMI = complexo médio-intimal; D = direito; E = esquerdo; M = masculino; F = feminino; DP = desvio padrão.

**Tabela 3:** Comparação quanto ao complexo médio-intimal por estado nutricional, sexo e lados direito e esquerdo das carótidas comuns.

**Comparação**	**Diferença estimada (mm)**	**Intervalo de confiança de 95%**	**p**
Sobrepeso/obesidade – Eutrofia	0,80	0,05-0,11	< 0,01
Lado D – Lado E	0,00	-0,01-0,01	0,94
Feminino – Masculino	-0,01	-0,04-0,02	0,47
Sobrepeso/obesidade – Eutrofia (Lado D)	0,08	0,05-0,11	< 0,01
Sobrepeso/obesidade – Eutrofia (Lado E)	0,07	0,04-0,10	< 0,01
Sobrepeso/obesidade – Eutrofia (Lado D – Lado E)	0,00	-0,01-0,02	0,60
Eutrofia – (Lado D – Lado E)	0,00	-0,02-0,01	0,67
Sobrepeso/obesidade – Eutrofia (Feminino)	0,06	0,02-0,10	< 0,01
Sobrepeso/obesidade – Eutrofia (Masculino)	0,09	0,05-0,14	< 0,01
Sobrepeso/obesidade (Feminino – Masculino)	-0,03	-0,07-0,02	0,21
Eutrofia (Feminino – Masculino)	0,00	-0,04-0,05	0,83

D = direito; E = esquerdo.

As estatísticas descritivas para espessura do CMI de carótidas comuns, de ambos os lados, mediadas no grupo com presença ou não de sobrepeso/obesidade, encontram-se expressas na Tabela 2. As crianças classificadas com sobrepeso/obesidade apresentaram CMI de carótidas comuns mais espessas que as do grupo eutrófico, com média de 0,49 mm no grupo sobrepeso/obesidade e 0,41 mm no grupo eutrófico (Tabela 2), com valor de p < 0,01. Não houve diferença entre as medidas do CMI de carótida comum direita e esquerda (p = 0,94). Os valores médios da espessura do CMI das carótidas comuns no grupo com sobrepeso/obesidade foram de 0,49 mm do lado esquerdo e de 0,5 mm do lado direito (p = 0,60), enquanto os valores da espessura do CMI no grupo eutrófico foram de 0,41 mm em ambos os lados (p = 0,67). Ao calcular as médias da espessura do CMI por sexo, os valores foram semelhantes (p = 0,47).

Para analisar a correlação entre o escore z do IMC e o CMI, foi proposto o coeficiente de correlação de Pearson (r), que quantifica a associação entre duas variáveis quantitativas. Observou-se um coeficiente de correlação de Person de 0,61, com significância (p < 0,01) e intervalo de confiança de 95% de 0,42-0,75. A Figura 2 expressa a forte correlação positiva dos sujeitos de pesquisa, de ambos os grupos, por grau de espessura do CMI das carótidas comuns, através de modelo de regressão linear com efeitos mistos^23^.

**Figura 2:** Correlação entre escore z do índice de massa corporal (IMC) e complexo médio-intimal (CMI). Coeficiente de correlação de Pearson (r) de 0,61 [intervalo de confiança (IC) de 95% = 0,42-0,75].

## DISCUSSÃO

Desde a fase pré-púbere, as crianças com excesso de peso são expostas a fatores de risco cardiovascular, que conduzem à disfunção endotelial e contribuem para o aumento da espessura do CMI da carótida comum^24^. Em 2010, Juonala et al.^25^ apontaram, a partir de análise de dados de quatro grandes estudos populacionais e de coorte (o *Muscatine Study*, o *Cardiovascular Risk in Young Finns Study*, o *Bogalusa Heart Study* e o *Childhood Determinants of Adult Health Study*), que a exposição aos múltiplos fatores de risco dos noves anos de idade em diante seria preditiva de aterosclerose subclínica na idade adulta. Assim, eles sugerem que esses fatores, diretamente associados à morbimortalidade por doença cardiovascular, sejam mensurados a partir dos nove anos de idade.

A espessura do CMI é um marcador subclínico de aterosclerose bem estabelecido, podendo também indicar futura doença cardiovascular^26^. O presente estudo confirma os achados ultrassonográficos das alterações do CMI em crianças com sobrepeso/obesidade (Tabelas 2 e 3), quando comparadas ao grupo controle, independentemente de sexo e lado direito ou esquerdo de carótidas comuns. Um estudo de caso-controle realizado na Bélgica por Beauloye et al.^27^ utilizou a ultrassonografia para avaliar a espessura do CMI em indivíduos saudáveis obesos e não obesos, com idade entre 8 e 18 anos (média de 12,7 e 13 anos, respectivamente). A média dos valores de espessura do CMI mostrou diferença estatística de 0,438 mm nos controles para 0,470 mm nos obesos (p = 0,0031). Apesar de a idade média ser superior à do presente estudo, os resultados foram semelhantes no que se refere à diferença significativa da espessura do CMI entre os dois grupos. É importante salientar que, considerando os dois estudos como uma sequência, pode-se observar que as alterações carotídeas se iniciam precocemente e persistem durante a infância e a adolescência. Os resultados são similares aos de outros estudos que relataram espessura do CMI de crianças e adolescentes obesos maior quando comparados ao grupo controle^28^^,^ 29. Fang et al.^30^ avaliaram 86 crianças e adolescentes obesos de ambos os sexos distribuídos em dois grupos, sendo 23 crianças obesas com síndrome metabólica (média de idade: 10,9 ± 1,6 anos) e 63 crianças obesas sem síndrome metabólica (média de idade: 10,5 ± 1,6 anos). O grupo controle foi composto por 22 crianças e adolescentes saudáveis não obesos (média de idade: 11,1 ± 2,1 anos). Os grupos de obesos sem síndrome metabólica e com síndrome metabólica apresentaram aumento da espessura do CMI quando comparados ao grupo controle.

O presente estudo apresenta algumas limitações. A principal delas refere-se ao caráter transversal da coleta de dados, que impede o direcionamento dos resultados para relações de causa e efeito. Também o número total de crianças avaliado foi pequeno, apesar de suficiente para as análises estatísticas. Por fim, foi utilizada uma amostra de conveniência, o que impede a extrapolação dos resultados para a população de crianças dessa faixa etária.

Os resultados analisados permitem concluir que as crianças com sobrepeso/obesidade apresentaram CMI das carótidas comuns mais espesso (0,49 ± 0,07 mm) quando comparadas às crianças eutróficas (0,41 ± 0,05 mm) da mesma faixa etária e de ambos os sexos (p < 0,01), resultados também observados por outros autores^31^^-^ 33. A principal contribuição do presente estudo refere-se à faixa etária, uma vez que são muito raros estudos ultrassonográficos do CMI nessa idade. Os resultados apresentados poderão, seguramente, contribuir para o melhor entendimento da evolução do processo aterosclerótico em crianças com excesso de peso.
